# Health worker experiences of and movement between public and private not-for-profit sectors—findings from post-conflict Northern Uganda

**DOI:** 10.1186/s12960-016-0114-y

**Published:** 2016-05-05

**Authors:** Justine Namakula, Sophie Witter, Freddie Ssengooba

**Affiliations:** ReBUILD Consortium and Department of Health Policy Planning and Management, Makerere University, School of Public Health, P.O. BOX 7072, Kampala, Uganda; ReBUILD Consortium and Institute for Global Health and Development, Queen Margaret University, Edinburgh, UK

**Keywords:** Health workers, Public sector, Private not-for-profit, Uganda, Post-conflict, Attraction, Retention

## Abstract

**Background:**

Northern Uganda suffered 20 years of conflict which devastated lives and the health system. Since 2006, there has been investment in reconstruction, which includes efforts to rebuild the health workforce. This article has two objectives: first, to understand health workers’ experiences of working in public and private not-for-profit (PNFP) sectors during and after the conflict in Northern Uganda, and second, to understand the factors that influenced health workers’ movement between public and PNFP sectors during and after the conflict.

**Methods:**

A life history approach was used with 26 health staff purposively selected from public and PNFP facilities in four districts of Northern Uganda. Staff with at least 10 years’ experience were selected, which resulted in a sample which was largely female and mid-level. Two thirds were currently employed in the public sector and just over a third in the PNFP sector. A thematic data analysis was guided by the framework analysis approach, analysis framework stages and ATLAS.ti software version 7.0.

**Results:**

Analysis reveals that most of the current staff were trained in the PNFP sector, which appears to offer higher quality training experiences. During the conflict period, the PNFP sector also functioned more effectively and was relatively better able to support its staff. However, since the end of the conflict, the public sector has been reconstructed and is now viewed as offering a better overall package for staff. Most reported movement has been in that direction, and many in the PNFP sector state intention to move to the public sector. While there is sectoral loyalty on both sides and some bonds created through training, the PNFP sector needs to become more competitive to retain staff so as to continue delivering services to deprived communities in Northern Uganda.

**Conclusions:**

There has been limited previous longitudinal analysis of how health staff perceive different sectors and why they move between them, particularly in conflict-affected contexts. This article adds to our understanding, particularly for mid-level cadres, and highlights the need to ensure balanced health labour market incentives which take into account not only the changing context but also needs at different points in individuals’ life cycles and across all core service delivery sectors.

## Background

The conflict in Northern Uganda between the Uganda army and the Lord’s Resistance Army (LRA) rebel group lasted for 20 years (1986–2006). This resulted in loss of lives, mass displacement (within and outside the region) for ordinary people and the health workforce, discontinuation of social services and destruction of infrastructure such as roads, schools and health facilities [1–3]. At the end of the conflict, the health system was split into a functional camp-based health system run by international agencies and non-governmental organisations (NGOs), on the one hand, and a few government health facilities confined to towns or in protected areas on another [4]. The rest of the government-owned health facilities had been destroyed and remained dysfunctional without supplies. The private not-for-profit (PNFP) sector also played an important role in filling the gap in health service delivery, particularly in very remote areas and in times of epidemics such as Ebola and cholera [5, 6]. The Uganda Catholic Medical Bureau (UCMB), for example, owns 22 % of health facilities in Northern Uganda [7].

Since 2006, more than 85 % of internally displace persons (IDPs) living in camps are reported to have returned to their villages of origin or moved to transit/satellite camps closer to their homes [8]. Although this resettlement has been gradual, it has raised issues of access and equity as well as the need for health system and human resources for health reconstruction [9].

In Northern Uganda, reconstruction efforts are coordinated under the overarching framework of the Peace and Recovery Development Plan (PRDP 2007). These reconstruction efforts have included the strengthening of local government capacity and rehabilitation of critical infrastructure such as health facilities and roads [10]. However, there are still challenges for HRH which are common on post-conflict settings [11], such as poor skill mix, geographical imbalance and difficulties in attraction of health workers.

Public and PNFP sectors both play a significant role in health service delivery in post-conflict Northern Uganda. However, there is a risk that they compete with one another for a limited supply of qualified staff. With improvements in the terms and conditions in the public sector, the PNFP sector is perceived to be suffering from increased attrition, especially in key clinical cadres and at the hospital level [7].

The effects of health worker movement between sectors include the creation of inequities for the population served by the sectors and the weakening of health systems, as some sectors are left with fewer health workers [12, 13]. The situation can even be worse for post-conflict settings where the populations are concentrated in rural areas and where attracting and retaining staff can be particularly difficult [14–16].

Understanding the movement of health workers between public and PNFP sectors is vital to ensure that both are able to contribute to universal health coverage post-conflict and to health system resilience in the face of future conflicts, epidemics and other shocks. Few studies have focused on health workers’ experiences of different sectors over time, in conflict and post-conflict contexts.

This article has two objectives: first, to understand health workers’ experiences of working in public and PNFP sectors during and after the conflict in Northern Uganda, and second, to understand the factors that influenced health workers’ movement between public and PNFP sectors during and after the conflict.

## Methods

This was a phenomenological qualitative study which mainly employed the life history method to enable the research team to acquire in-depth information and to capture a trajectory of health workers’ experiences and movement between sectors across their careers and across the different phases of conflict.

The creation of time lines by the participants enabled their active participation in the research process and also iterative probing on key events (as perceived by individual health workers) and decisions made by health workers across their career paths and conflict phases.

### Site and sample selection

The study was conducted in the Acholi sub-region, Northern Uganda, in the districts of Pader, Gulu, Amuru and Kitgum—as these were the most affected by the conflict and contained more than 90 % of the displaced people. Twenty-six life history interviews were conducted with health workers who were purposively selected, based on district, sector (public and PNFP) and level of health facility (Table 1). Selected participants should have worked in the Acholi region for at least 10 years. This was because 10 years would enable us to capture their experiences during 5 years at least of conflict and 5 years post-conflict. The number of years worked also influenced the cadres and gender of health workers who ended up being included in the sample. The majority who qualified were women (19 females, 7 males). The distribution of interviews by district was seven for three of the districts and five for Amuru. The distribution across sectors was 17 in the public sector and 9 PNFP. All PNFP facilities included in the study were under the umbrella of the UCMB, as this is the largest PNFP sub-sector in the Acholi sub-region.

**Table 1:** Characteristics of participants

Characteristic	Average	Range
Age	42 years	30–60 years
Time spent working in the region	17 years	7–38 years^a^
Sex	23 % M, 77 % F	
Cadres	Clinical officers (15.38 %), nurses (57.68 %), nursing assistants (7.69 %), midwives (11.53 %), others (7.68 %)	
District	27 % Pader, 27 % Kitgum, 19 % Amuru, 31 % Gulu	
Sector	65 % public, 35 % PNFP	
Type of health facility	Hospitals (31 %), HC IV (15 %), HC III and II (46 %), others (8 %)	
Highest level of formal education	69 % O’Level^b^, 12 % A’level^b^, 15 % diploma, 4 % degree	

^a^One person was accepted with fewer than 10 years’ experience in one facility which lacked anyone meeting the selection criteria

^b^O’level is the British Ordinary Level certificate, normally sat around 16 years old; A’level is the Advanced Level certificate normally sat at end of secondary school, at around 18 years of age

### Data collection

Fieldwork was conducted in October 2012. A life history approach was adopted. During the interview, a horizontal line was drawn on a piece of paper, and with assistance from the interviewer, participants were encouraged to record key events and decisions in their lives along the line. Probes were made about the decision to become a health worker, training experiences, their employment history, why they had stayed or moved between jobs, their job satisfaction, experience of conflict, coping strategies during and after conflict and experience of different policies and future plans, including intentions to move. The life line was used as a tool to guide discussion, and at the end of the interview, it provided a visual representation/summary of key events, as perceived by the participant. The findings discussed in this paper will be focused on the movement of health workers between public and PNFP sectors and the factors driving their movement between the sectors during and after the conflict.

### Ethical approval

Ethical approval clearance was granted by the Makerere University School of Public Health Higher Degrees Research and Ethics Committee and by the Uganda National Council for Science and Technology and the Research Ethics Committee at the Liverpool School of Tropical Medicine in 2012.

### Data analysis

Data analysis was guided by the framework analysis approach of Ritchie and Spencer [17] and analysis framework stages provided by Ritchie and Lewis [18]. ATLAS.ti software version 7.0 was used to manage the data. Audio tapes were listened to and compared with notes taken during interviews to fill in the gaps in information that could have been left out or miss-recorded during note taking. Audio recordings were transcribed verbatim so that original meanings are not lost. Transcripts were read several times to get an overall picture, and then, recurring preliminary themes were identified and used to create codes.

A code book was generated, and data were then prepared for entry into ATLAS.ti by filing transcripts using identifiers such as district, current sector of employment, level of health facility, cadre and gender. Filed transcripts and codes were then uploaded into ATLAS.ti, and coding nodes were attached to quotations. ATLAS query reports were generated and printed out for each code and further familiarised to identify more sub-themes. Similar sub-themes were merged together to create themes, whereas in some cases, sub-themes were created. These themes were further entered into pivot tables with each respondent anonymised as a personal identification (PID) number. Finally, quotations that epitomised the emerging themes were identified and agreed upon by the research team. Selected quotes were labelled according to PID, gender, sector of employment and district. Life lines were used as guides during interviews and also to aid analysis of patterns of movement. The life lines are not included in the article for confidentiality reasons, as the information in them can reveal individual identities.

### Results

We present findings which follow the logic of the life history. We first discuss initial training experiences, as these are presumed to influence subsequent decisions to stay or leave a sector. We then present their subsequent working experiences during and after the conflict, focusing on their experiences of differences between the sectors. Next, we present factors reported to influence their decision to move or not between public and PNFP sectors as well as their future intention to move.

### Health workers’ experiences of public and PNFP sectors at initial training

A total of nine training institutions were mentioned by participants as places where they had their first (initial) training in medical skills, mostly during the conflict period. The majority of the participants had their initial training in nursing schools within missionary (PNFP) hospitals located within the greater Northern Uganda. Fewer participants had their training in government-owned training institutions located within the greater Northern Uganda region or outside the region.

The themes discussed under training experiences related to management of training facilities, quality of teaching experienced, workload and incentives.

#### Management of training facilities

In relation to management, there were reported differences between the PNFP sector and public sector. Compared to the public sector, the training institutions under PNFP were reported to be characterised by more strict management styles reflected in various restrictions.

These restrictions were related to movement outside the hospital facility, having sexual relationships and general behaviour. These restrictions often created tension as well as dissatisfaction among the students at the time. Non-compliance to the restrictions could attract punishments such as suspension or dismissal.

[…] as students during that time, we were being restricted not to go out or any here beyond the hospital. […] you are not allowed to go out unless you have been permitted and you are supposed to go out once a month. PID 24 Male, PNFP, Pader

[…] I tell you the principal was too strict! We used to have few boys but now they are many. So there was a lab where boys and girls met, as you know the adolescent age, they could relate […]. When you see her (the principal) coming at a distance you must run […]. PID 11 Female (but trained in the PNFP sector), Public, Kitgum

#### Quality of training

Health workers in PNFP and public institutions reported good tutors during their initial training. Health workers who trained in PNFP institutions reminisced about the good and present tutors, under whose close supervision they worked as students. Experiences of being supervised by expatriates were also common among participants in this category. However, health workers who went to the public training institutions reported that sometimes the tutors were unavailable and this affected their future practice as they had missed out on some components in the initial training.

During the training time, it was very good because we had a British tutor called miss JXX who was very good, very motherly and gave us all the basics in the training, i.e. the skills, knowledge and during practicals she was with us, what she has taught she could follow us up to the ward and say that you are performing the real thing on the ground. PID 12 Female, PNFP, Kitgum

My experience in XX (public) training school of Nursing, first we didn’t have good tutors […] you had to struggle on your own so I had my sister who had already completed in Training school xx [a PNFP institution], […] she helped me with her notes […] They could rarely come to teach us […]. PID 9 Female, Public, Kitgum

Health workers reported that they were exposed to clinical practice during their initial training, regardless of whether they went to a PNFP or public training institution. However, those who attended the PNFP training institutions indicated that they had more exposure.

[…] otherwise when you join for the first three weeks, you are taught many things before you can go to the ward. So you end up with a lot of experience because they have a lot of materials. PID 11 Female, Public, Kitgum

[…] working in a missionary hospital is really good because there you are exposed to so many cases, you see cases and then they motivate you in very many ways because you see how it is done in reality. PID 16 Female, Public (but trained in the PNFP sector), Amuru

#### Workload during training

Unlike their colleagues who studied in public institutions, health workers trained at PNFP facilities reported a high workload as students. They attributed this to effects of the war (increased number of war casualties) and epidemics, particularly Ebola around 2000–2001. For some, such experiences broadened their knowledge but at the same time resulted in fear and anxiety. However, perseverance, counselling and support from tutors played an important role in reducing panic.

The training was o.k. only that because of the insurgency […] we had a lot of experience you [could] find a full track of soldiers being brought with injuries so the students have to be taken on the ward to work on them, we had too much work that time though we were still students but there was no option we had to work on the victims. PID 15 Female, Public, Gulu

During those days, during Ebola outbreak [2000–2001], it was not easy because we had many patients […] it was such a scary thing for us. In most cases, we were working on rebels themselves. But we had our teachers who were counsellors and so the fear disappeared […]. PID 2 Female, PNFP, Amuru

#### Incentives received during training

During their initial training, health workers received a number of incentives ranging from basic necessities such as food, sugar, washing soap and free medical treatment as well as some pocket money. Non-financial incentives, for instance, provision of good food, were common among those that went to PNFP schools. On the other hand, financial incentives such as pocket money were mentioned by a few of those who attended public training institutions, particularly in the 1970s but no longer in the late 1990s. With exception of those who received scholarships from PNFP facilities to convert from nursing aide to nursing assistant, financial incentives were not mentioned by those trained in PNFP establishments.

At the end of the month they could give us [all students] sugar, and also a bar of washing soap, some little money,[…] it was 12,000/=[ in 1978] [[…]. PID 23 Female, PNFP, Pader

[…] in 1998, I was XX public training institution sponsored by the HSSP programme at the district. It was quite hard […] my father could send some little money for up keep, the institution[public] used not to give us any allowance so you were to find your level. PID 26 Female, Public, Pader

From 1999 up to 2002 I trained from XX PNFP institution as a Nursing aide. […] life was so fine because, we were being fed as students. PID 18 Female, PNFP, Amuru

### Health workers’ subsequent job experiences in PNFP and public sectors

Health workers’ subsequent experiences working in the PNFP and public sectors were mainly differentiated by working conditions. Working conditions may include many parameters, but participants focused on management and leadership, workload and working hours, and incentives.

#### Management and leadership

Health workers in the PNFP sector encountered more restrictions during their subsequent job postings compared to those who worked in the public sector. Whereas at the initial training, restrictions in the PNFP sector concentrated around relationships, movements outside the hospital environment and general behaviour, for the subsequent jobs, the previous restrictions still held but new ones were reported to be introduced. These new restrictions were related to zero tolerance on dual practice (particularly for medical work), whereas within the public sector, there were regulations but some flexibility. Additionally, compared to the public facilities, the PNFP facilities showed a continued strictness on time management.

Experiences of working under the guidance and leadership of expatriates, who were mainly missionaries from Italy, were reported by health workers who worked in both sectors particularly in the conflict period, with little mention in the post-conflict period. Such experiences reportedly enabled the health workers to learn values such as hard work, quality assurance, empathy, appreciation and persistence which they took on throughout their career.

[…] So here [at PNFP facility] we come right in time but in government whether you come on duty [in time] or not your salary will be there you see? […]. Some people [health workers] are running small business like selling these second hand clothes. […] after work […] but what they don’t want is selling drugs. PID 12, Female, PNFP, Gulu

I have a drug shop to help me supplement my income […] But there are lots of restrictions [in public sector], the district comes with their policies and the government with their own also that we should not be having these drug shops or clinics. PID 25, Male, Public, Pader

This hospital (public facility) was being strongly supported by Italians by that time[of the war] but eventually when they were leaving […] they had nurtured the African doctors to take over […] and these remained wonderfully working but the working conditions continued to be very hard. PID 6 Female, Public, Kitgum

There was reported flexibility related to leave entitlement in the conflict period, particularly for health workers who had survived abduction to enable them to try to overcome these traumatic experiences before resuming work. Study leave was also granted for public and PNFP health workers. However, health workers reported differences across sectors in relation to implementation of leave entitlement with the public sector being perceived as having better leave terms. These differences caused dissatisfaction and influenced intention to migrate from one sector to another.

When you go for study leave they give us 40,000/= as pocket money per month but in the government they give full salary. So if you have a family like mine you cannot do anything with that 40,000/= per month […]. PID 24 Male, PNFP, Pader

#### Workload

Workload was reported to have increased in both sectors during the conflict. However, compared to the public sector, PNFP facilities had heavy workload partly due to their functionality in the conflict period and during responses to epidemic outbreaks such as Ebola and cholera in relation to public facilities. Health workers who worked/reported in public facilities located within internally displaced person (IDP) camps also experienced increased workload. However, after the conflict subsided, workload was perceived to have remained higher in the PNFP sector within and outside the Acholi sub-region, and this was one of the reasons cited for intention to migrate from the PNFP to the public sector. The positive side of experiencing high workload was that the health workers felt able to work with minimal supervision in their subsequent job postings along their career.

During those days, during Ebola outbreak, it was not easy because we had many patients […]. PID 2 Female, PNFP, Amuru

Yeah, during […] the war times […] around June-august 2002 […]we would work all night, we were having casualties, two lorries […] we started working from 6.00 p.m. to around 4.00 a.m. […] then the following day from 11.00am to midnight […]. PID 24 Male, PNFP, Pader

The number of patients was overwhelming, because all these five refugee camps, I think about 13,000 people were being served by 2 health centres […] too many injuries. PID 25 Male, Public, Pader

[…] then I worked in [Outpatients’ department] OPD of Atapara missionary hospital. Eeeh! […] to be sincere we could work […] you know being a missionary hospital in that area […] we had very many patients […]. PID 16 Female, Public, Amuru

#### Salaries, allowances and incentives

Low salary was reported by health workers in both sectors during the conflict and during subsequent jobs, although complaints about it became more common in the later career stages when family needs and expectations were higher. Absence of salaries during the conflict, delays in receiving salary and complaints of being deleted from the payroll were only reported by public health workers. PNFP health workers seemed to seek comfort from the other non-financial benefits and by running non-drug-related businesses to bridge the gap between salaries and living costs. Public health workers coped by getting support from the communities where they worked, brewing alcohol and any similar small businesses, undertaking dual practice and receiving allowances from NGOs especially for outreach work. Only one participant who worked in the PNFP sector attempted to open a drug shop as a supplement to income but this was later closed. Outreach work in communities was not mentioned by PNFP health workers.

The work was okay, but the problem was no money, […] at that time I was not on salary. They were giving us some allowances when we go for outreaches, and the NGOs who were on the ground would motivate us plus risk allowance during the period of Ebola [2000–2001]. PID 12 Female Public, Gulu

[When I finished training and was retained in Lacor] it [the salary] was little- I think it was 150,000, 170,000. […] the good thing […] they had given us accommodation and were paying for our electricity and we had water in the hospital […]. PID 11 Female Public, Kitgum*.*

In XX PNFP facility, we started with something small in terms of salary, but they have increased and I can’t really compare because I don’t have at hand what my colleagues in the government have. But the only good part is that normally our salaries don’t delay, they pay us really in time. PID 19 Female PNFP, Gulu

[…] posted to XX public Health centre in 2000 but no salary for 6 months. […] Surviving on allowances from MSF Spain. PID 16 Female Public, Amuru

In relation to mainstream (nationwide) incentives meant for all health workforces, only health workers in public facilities received ‘hard to reach’ and consolidated allowances (monetized benefits for housing, meals at work transport, etc.). The ‘hard to reach’ allowance was provided as affirmative action to those in remote and conflict-affected areas (from 2010), whereas the consolidated allowance was meant for all civil servants (from 1996). Some health workers in PNFP also benefited from monetary allowances in addition to salaries. These were from temporary sources such as NGOs and short-term projects being implemented at PNFP facilities.

[…] right from the first, second and all the jobs that I was being transferred to, I still received the hard to reach allowance. It’s of recent because of decentralization that they have chopped off yet this place is still a hard to reach place […]. PID 25 Male Public, Pader

[…] we do not get hard to reach allowance […]. PID 18 Female PNFP, Kitgum

That is why the rate of retention of staff in NGOs is less because we work minus those consolidated lunch allowances […]. We are depending on donors. PID 12 Female, PNFP, Gulu

Unlike in the public sector, incentive packages in the PNFP sector were reported to be predominantly non-financial. These included free/subsidised housing, free water, free/subsidised medical care and scholarships for upgrading, among others. However, differences in the incentive packages were reported in different UCMB facilities within the same region, particularly in relation to housing and access to medical care. Some working in PNFPs within Gulu received a monetary allowance package for work in the remote facilities affiliated to the PNFPs, although this varied according to cadre.

Yes, in comparison with XX PNFP hospital, where the staff get free medical treatment here, we are given a pre-paid card worth 80,000/= which we can use for treatment […] If you use up all the money, then you have to top up with your own money. PID 24 Male PNFP, Pader

We have been given accommodation, there is light (power), there is water and if you are not well, treatment is free inclusive of your children, husband and parents. We are provided with that free medical service. PID 12 Female PNFP, Gulu

### Other factors influencing health workers’ movement between public and PNFP sectors

As a result of insecurity in the Acholi sub-region, health workers (regardless of the sector they were working in) were migrated to areas which were perceived as being safer. In some cases, this led to a shift in their sector of employment.

We fled to Angal Hospital [in Nebbi district, West Nile sub-region] from Kalongo due to Insurgency. PID 7 Female, PNFP, Kitgum

Given that the [war] situation was horrible, I abandoned the job at Kitgum hospital and went to stay in Kampala for 2 years […]. PID 26 Female, Public, Pader

[…] until 1986 when […]the rebels over ran the place […] we ran together with the soldiers, […] I stayed in the village a bit because coming to town was a bit of a problem […], […] I went to town in 1987 and started working in Kitgum general hospital. PID 22 Male, Public, Pader.

The most common category of inter-sectoral movement during and after the conflict was from the PNFP to the public sector. The reasons for movement from the PNFP to the public sector during the conflict were mainly retention packages within the public sector, particularly favourable leave entitlements, pension benefits and a retirement package, all of which were perceived as non-existent in the PNFP sector. The other reasons were related to perceived flexibility of work hours within the public sector and perceived reduction in workload compared to that in the PNFP sector.

[…] in 2002, I got a vacancy at Kitgum hospital [public] on contract under an NGO. PID 26 Female, public, Pader

What made me leave that job [in PNFP] in 2001 was because […] government gives […] pension when you reach retirement age […]. Also […] when you lose your mother, missionary hospitals give you only two days. […] but in government […] you get a one week for you to at least mourn and come back when you are fresh. PID 11 Female, Public, Kitgum

[…] my first job [in PNFP] was interesting. […] however, the work was too much and […] no freedom, you wouldn’t have any hour of doing your own things because every time you are supposed to be on duty! [..] Life became very difficult and so in [1996] I applied to government service commission when they advertised, passed the interview and got the job […]. PID 16 Female, Public, Gulu.

One of the health workers decided to move from the PNFP to the public sector because they felt the skills they had acquired during one of the short courses could not be exercised in the PNFP setting. Another left the sector due to conflicts with fellow health workers.

I worked at XX PNFP Hospital for 20 years without transfer. Although I was sponsored [by the PNFP hospital] for a public Health course for one year […] promoted on return […] and paid well. […] What made me come to Local Government [in 2011] was because […] I was not practicing my new skills as a public health nurse [at the PNFP facility]. […] therefore I applied to local government. PID 13 Female, Public, Gulu

[…] at the PNFP facility[where I worked] there was lot of conflict in the nurses’ quarters, so I first abandoned the hospital accommodation and went to stay outside[the hospital] but then I ended up spending almost all my salary on transport and so I decided to leave PNFP to government. So, [in 2008] I applied in 2008 and got the government job. PID 19 Female, Public, Gulu

Only three participants mentioned that they had moved from the public to the PNFP sector during the conflict; they highlighted insecurity and the need to reunite with their families as the main reasons. None had shifted to the PNFP sector post-conflict.

War broke out and there was massive displacement in 1987–1988. We moved from Kalongo to Adilang sub-county in West Nile and [in 1988] that was when I joined local government. PID 20 Female, Public, Pader

[…] in 1999, I left Kitgum general hospital [public] and moved to Kalongo [PNFP] because I was uncomfortable I had been separated from my husband and family and wanted to be with them. PID 22 Female, PNFP, Pader

### Reasons for not moving between sectors

In some cases, health workers never moved between sectors. Some health workers were rotated within departments in the same facility of the same sector in the same district, whereas others were not rotated but merely promoted. On average, these health workers worked in the same facility for 27 years.

An almost equal number of health workers stayed in either the PNFP or the public sector without changing sectors. A number of reasons were mentioned for not moving. Those who stayed working in the PNFP sector for long highlighted the role of bonding after sponsorship, often referred to as ‘being detained at training institution after completion of upgrade studies’. Other reasons highlighted for not switching sectors included good working relationships, limitations due to family obligations and availability of free health care for them and their families. Others did not give any reasons but merely noted ‘staying in that sector’ as a key milestone in their career.

[…] after exams in November they [hospital] also called me back to come and work here. […] if they [the hospital administration] sponsor you, you have to comeback straight here and work. You cannot go anywhere […]. PID 24 Male PNFP Pader

[…] There was [and there still is] free health care for staff and family. PID 1 PNFP, Kitgum

For those who had stayed for a long time within public sector facilities, their life lines indicated that they were motivated to stay due to the promotions and frequent short trainings they were given within the same facility. Other health workers attributed their stay in the public sector (during the conflict) to their dedication and commitment to their jobs and a sense of giving back to the sector for having funded them for their initial training.

[…] everything was paid by the Ministry of Health and I was very grateful. That is why I am very happy and I am serving here in the village because without that sponsorship from the ministry, I would not have managed. […] that is why I have continued and will never withdraw from the district because it was a nice beginning, a foundation. PID 9 Female Public, Gulu

[…] So war came and got us here and of course the war scattered most of the staff, […] People had to run to save their lives […] I had the spirit that I am better off dying from my workplace and that encouraged me to stay. PID 6 Female Public, Kitgum

### Future intention to move

The health workers were also asked about their intention to move from one sector to another in their later career stages and the reasons why. Although there was intent to move from PNFP to public and within both sectors, none of the health workers expressed an intention to move from public to PNFP.

The reasons given by the health workers who intended to move from the PNFP to the public sector included perceived better job security in the public sector, desired to benefit from mainstream public incentives (such as hard to reach allowance and pension) and perceived high workload in some PNFP facilities. Other reasons were as follows: limited opportunities to attend workshops within the PNFP sector, perceived high salary in the public sector and a desire to work in a sector with perceived ‘flexible’ rules on dual practice and flexibility related to leave entitlement.

[…] there is no job security here. Staff is on an annual contract. PID 1 Male PNFP, Kitgum

Sometimes you think, ‘should I try to go to government?’ […] you have a lot of responsibility and what you earn is little so that is a little bit demoralizing. PID 12 Female PNFP, Kitgum

[…] we have a lot of work. […] If you‘re in government, you have a very big chance of going for workshop […]. PID 24 Male PNFP, Pader

## Discussion

The study utilised the life history tool which was found effective in understanding health workers’ embedded experiences of sectors of employment during and post-conflict. The health workers involved were largely mid-level and female, which reflects the composition of the health workforce in this area. A recent study found that 77 % of health workers in Northern Uganda were female [19].

Our findings, summarised in Table 2, indicate that conflict affected health workers’ experiences of the PNFP and public sectors. Additionally, the study highlighted a relationship between health workers’ experiences of public and PNFP training and working and their movement or intention to move between the two sectors. This study adds to the limited literature on health workers’ experiences and movement between the PNFP and public sectors, particularly in fragile and post-conflict settings.

**Table 2:** Summary of findings

Themes	Public sector	PNFP
Training experiences		
Management		Stricter rules
Quality of training	Good tutors but some absences More limited clinical exposure	Good and present tutors Good exposure to clinical experience
Workload		High workload
Incentives	Wider range of incentives, including financial	Limited (mainly non-financial) incentives
Working experiences		
Management and organisational culture	Perceived greater flexibility about leave arrangements Exposure to expatriate (NGO) staff—appreciated—especially during conflict	More restrictions (e.g. on dual practice and time management) Exposure to expatriate (missionary) staff, with positive effects on learning, especially during conflict
Workload	High workload in IDP camps	High workload during and after conflict—but helps to maintain skills
Incentives	Low salaries, especially important later in middle of life cycle; irregular or absent during conflict; various coping strategies described Benefit from monetary incentives such as consolidated allowance and hard to reach allowance	Low salaries, especially important in middle of life cycle; various coping strategies described Only access short-term, externally funded monetary incentives Package of non-financial incentives, which vary across facilities
Reasons for moving, staying and factors influencing future intentions		
Attraction	Better overall package—leave, pension, allowances, higher salary Flexibility on leave and dual practice Lower workload Able to exercise skills better Better job security More access to training	Bonding (but sometimes perceived as detention) Good working relationships and ties of family obligation Availability of free health care for them and their families
Retention	Loyalty to sector which trained you Promotion Frequent short trainings	Strict rules on dual practice/inflexibilities on leave Perceived high work load No job security (mainly contractual) Limited access to training

The findings highlight that the PNFP sector plays a dominant role in initial training, at least in the Acholi region. This is because it owned the majority of the training institutions in the region and attracted many health workers to its schools due to incentives provided, ways of teaching and practical experiences. This and related bonding practices encouraged some workers to stay long term in the PNFP sector; however, some health workers perceived bonding as coercive and referred to it as ‘detention’.

During the conflict, the PNFP sector remained more functional, including in terms of supporting staff with pay. Although workload was high, the sector was able to retain staff, but when the public sector re-established itself post-conflict and was able to offer better terms and conditions, the competition for staff became more intense.

The public sector was boosted in the post-conflict phase due to increased investments under the PRDP, consolidation of allowances and introduction of hard to reach allowances. The salaries also became more regular while pension continued to be provided. Our findings suggest that retention within the PNFP sector has had to rely on more personal factors, such as loyalty and family ties, while many still working in the PNFP sector express the intention to leave, if circumstances permit.

The PNFP sector has endeavoured to mitigate the problem through a number of strategies which include more recruitment, attempts to increase salaries, having health workers seconded by the government and training of managers to improve their skills [20]. Nevertheless, the extent to which both financial and non-financial interventions helps manage movement from the PNFP to the public sector cannot easily be ascertained [12].

The study also found that incentive structures influence the migration of health workers differently at different stages of their careers. For example, the PNFP sector has a good system for training to the extent that in some literature it has been referred to as the ‘internship centre’ [7]. However, in the later stages of their careers, health workers aspire to pensionable jobs, security and monetary benefits as opposed to in-kind benefits. The tendency is to move to the public sector is to seek these benefits. This needs to be taken account of when planning for stabilisation of the workforce in both sectors. For instance, the PNFP sector may have to consider establishing pensionable jobs and monetized benefits for cadres with increasing family responsibilities. Lessons can be learnt from countries like Zambia and Ghana where the government provides salaries and pensions for both the public and PNFP sectors [21].

Our findings are consistent with some previous studies in Uganda [20, 22–24]. However, some have argued that the total remuneration package for PNFPs is by far better than that of the public sector, particularly for doctors, although the biggest portion comprises non-financial incentives [20]. In other African countries such as Tanzania, movement between public and PNFP has also been triggered by perceived better payment and benefit packages in the former [25–27]. In Namibia, movement to the public sector was attributed to the presence of fringe benefits and better conditions of service [28]. Salary differentials have also been found to affect health worker migration and retention between sectors in Zambia, Ghana, Malawi and Ethiopia [13]. In general, however, studies tend to find better pay and conditions in the PNFP sector relative to the public [29], which is the converse of our findings for Uganda.

The general expectation, or hypothesis, that the PNFP sector will be characterised by better pay and working conditions (often with more international support), offset by stricter management and sustained by more altruistic behaviour [29], is only partially supported by our evidence. Certainly in relation to terms and conditions, the PNFP sector is no longer competitive in Northern Uganda. Workplace factors which would enhance loyalty are also less effective than might have been expected, with some management in the PNFP sector particularly experienced as hierarchical and unsupportive. Some of Herzberg’s motivators [30], such as feeling recognised and being given promotion as reward for achievements, are not being sufficiently deployed to offset these difficult terms and conditions.

The study was conducted in four districts of the Acholi sub-region and with a selection of health workers who met the selection criteria of ‘at least 10 years and above’; hence the study findings may neither be generalised to all health workers within the region nor to the rest of Uganda. This was a positive deviance study in that it only focused on those who stayed in the region and not those who left the region. The study was also based largely on the experiences of mid-level cadres, as they were the ones who had stayed working in the region over time. The largely female composition of the workforce influenced the gender balance of the final sample. No private sector staff were interviewed as provision of private health care is limited in remote rural areas of Northern Uganda. Additionally, given the limited space, the paper does not cover discussion of difference between the sectors in terms of motivation to join the profession, although these may have a bearing on subsequent experiences and choices.

## Conclusions

The study has highlighted that health worker experiences of and movements between sectors are dynamic and complex, affected by contextual changes, such as conflict, life cycle issues for health workers and also policy changes, such as changing allowances. Incentive policies to attract and retain health workers need to take these into account. In post-conflict areas like Northern Uganda where the PNFP sector continues to play an important service delivery role, attention needs to be paid to making their terms and conditions more competitive relative to the public sector. Interventions to stabilise numbers of health workers, particularly the nurses and midwives, to match the needs at all levels of the health system in both sectors require comprehensive and participatory development and assessment.
